# ecDNA replication is disorganized and vulnerable to replication stress

**DOI:** 10.1093/nar/gkaf711

**Published:** 2025-07-31

**Authors:** Jedrzej J Jaworski, Pauline L Pfuderer, Pawel Czyz, Gianluca Petris, Michael A Boemo, Julian E Sale

**Affiliations:** Division of Protein & Nucleic Acid Chemistry MRC Laboratory of Molecular Biology, Francis Crick Avenue, Cambridge CB2 0QH, United Kingdom; Department of Pathology, University of Cambridge, Tennis Court Road, United Kingdom; Cancer Research UK Cambridge Centre, Li Ka Shing Centre, Robinson Way, Cambridge CB2 0RE, United Kingdom; Department of Biosystems Science and Engineering, ETH Zurich, Klingelbergstrasse 48, Basel 4056, Switzerland; ETH AI Centre, ETH Zurich, Andreasstrasse 5, Zurich 8092, Switzerland; Division of Protein & Nucleic Acid Chemistry MRC Laboratory of Molecular Biology, Francis Crick Avenue, Cambridge CB2 0QH, United Kingdom; Wellcome Sanger Institute, Wellcome Genome Campus, Hinxton, Cambridge CB10 1SA, United Kingdom; Department of Pathology, University of Cambridge, Tennis Court Road, United Kingdom; Department of Genetics, University of Cambridge, Downing Street, Cambridge CB2 3EH, United Kingdom; Division of Protein & Nucleic Acid Chemistry MRC Laboratory of Molecular Biology, Francis Crick Avenue, Cambridge CB2 0QH, United Kingdom; Wellcome Sanger Institute, Wellcome Genome Campus, Hinxton, Cambridge CB10 1SA, United Kingdom

## Abstract

Extrachromosomal DNA (ecDNA) is a critical driver of cancer progression, contributing to tumour growth, evolution, and therapeutic resistance through oncogene amplification. Despite its significance, the replication of ecDNA remains poorly understood. In this study, we investigated the replication dynamics of ecDNA using high-resolution replication timing analysis (Repli-seq) and DNAscent, a method for measuring origin firing and replication fork movement, that we applied to both bulk DNA and to ecDNA isolated with FINE (Fluorescence-activated cell sorting-based Isolation of Native ecDNA), a new method for isolating, chromatinized ecDNA without DNA or protein digestion. We demonstrate that ecDNA in the COLO 320DM colorectal cancer cell line exhibits largely asynchronous replication throughout the S phase, contrasting with the conserved replication timing of the corresponding chromosomal DNA in RPE-1 cells and the chromosomally reintegrated ecDNA in COLO 320HSR. Replication origins on ecDNA are redistributed, and replication forks exhibit reduced velocity and increased stalling. Under replication stress induced by hydroxyurea treatment, ecDNA replication is further compromised, leading to altered origin activation, reduced fork velocity and eventual ecDNA depletion from cells. Our findings reveal fundamental differences in the replication dynamics of ecDNA, providing insights that could inform the development of therapies targeting ecDNA-associated oncogene amplification in cancer.

## Graphical Abstract

**Graphical Abstract:**
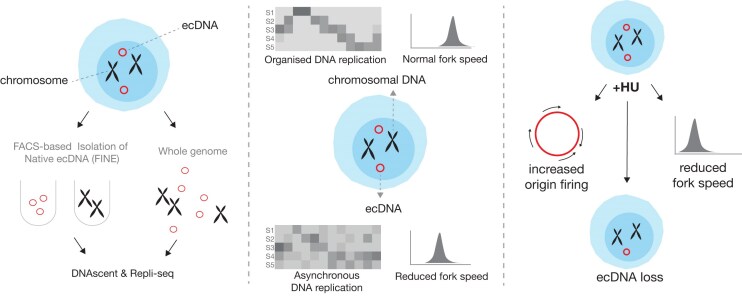

## Introduction

Oncogene amplification is a critical driver of cancer progression, contributing to tumour growth, evolution, and resistance to therapy. Oncogene amplifications arise from copy number increases which may be harboured on linear chromosomes, often observable as homogeneously staining regions (HSRs) in metaphase chromosome spreads [1, 2], or on extrachromosomal DNA circles (ecDNA) [2, 3]. ecDNAs, which can be present in hundreds of copies, are small nonchromosomal chromatin bodies [4] averaging between 1 and 3 Mb in size [5] and have been detected in ∼17% of all cancers [6].

ecDNA populations may be heterogeneous in terms of size and structure and may contain several copies of an oncogene in a single molecule [7–10]. In addition to oncogenes, ecDNA molecules often contain immunomodulatory genes [11–13], which contribute to the aggressive behaviour of ecDNA-containing tumours. ecDNA-based oncogenes may also be more highly transcribed even when adjusted for their high copy number [8, 9, 14] and this has been linked to the open chromatin and clustering observed in ecDNA molecules. Although this effect has been observed in some cell lines, including in the extensively studied colorectal carcinoma cell line COLO 320DM used in the present study, it may not be universal in all cell lines containing ecDNA [15, 16].

Unlike canonical chromosomes, ecDNA lacks centromeres, leading to its uneven segregation during cell division [17, 18]. This unique flexibility of ecDNA contributes to the rapid evolution of tumours by promoting intratumour genetic heterogeneity [18] allowing cancer cells to rapidly adapt to selective pressures, including therapeutic interventions [18–22]. Consequently, the presence of ecDNA is associated with poorer outcomes, including reduced survival rates compared to tumours without such amplifications [6, 11].

Despite the recognized importance of ecDNA in cancer, much remains unknown about the dynamics of DNA within these molecules. While ecDNA clearly replicates [3, 23], and likely only once per cell cycle during S phase [24], details such as the precise timing of replication, the distribution of replication origins, replication fork velocity, and the frequency of replication stalling remain poorly understood. For instance, it is unclear whether the replication origins and replication timing found in a chromosomal segment are preserved when present in ecDNA. Moreover, ecDNA is characterized by elevated replication stress [14], and its loss appears to be linked primarily to a further increase in this stress followed by micronuclei-mediated ecDNA elimination, particularly following treatment with hydroxyurea (HU) [25–28]. A more complete understanding of ecDNA replication may enable the development of strategies to control ecDNA copy number and, consequently, oncogenic drive in certain cancers.

To address these questions, we combine assessment of the replication timing programme using Repli-seq [29, 30] with DNAscent [31], a method for determining the velocity and direction of the nascent DNA synthesis in single DNA molecules, applying Oxford Nanopore Technologies (ONT) long-read sequencing of COLO 320DM cells and matched ecDNA-negative controls, the tissue- and patient-matched colorectal cell line COLO 320HSR and the untransformed, karyotypically stable, diploid cell line RPE-1. Further, to directly detect replicating ecDNA, we introduce FINE [Fluorescence-activated cell sorting (FACS)-based Isolation of Native ecDNA], a method for isolating largely intact, chromatinized ecDNA without requiring DNA or protein digestion. We show that replication timing programme in ecDNA is significantly disrupted. Further, replication in ecDNA appears more sensitive to stress induced by depletion of ribonucleotide pools with HU than chromosomal DNA, which leads to replication stress-induced ecDNA loss.

## Materials and methods

### Cell culture

Human colorectal adenocarcinoma cell lines COLO 320DM and COLO 320HSR were obtained from the American Type Culture Collection (ATCC) and maintained in RPMI-1640 medium (Gibco) supplemented with 10% (v/v) fetal bovine serum (FBS; Sigma–Aldrich). hTERT-immortalized retinal pigment epithelial cells (RPE-1) were also sourced from ATCC and cultured in a 1:1 mixture of Dulbecco’s modified Eagle’s medium and Ham’s F12 (Gibco) supplemented with 10% (v/v) FBS. All cell lines were incubated in a humidified atmosphere of 5% CO_2_ at 37°C. Cell lines were regularly tested for mycoplasma contamination.

### Metaphase chromosome spreads

Cells were arrested in metaphase by treatment with either 0.1 μg/ml KaryoMAX Colcemid solution in phosphate-buffered saline (PBS; Gibco) for RPE-1 cells, or 0.4 μM nocodazole (Sigma–Aldrich) for COLO 320DM and COLO 320HSR cells, for 3 h at 37°C. After arrest, cells were washed with PBS and gently resuspended in a pre-warmed 75 mM potassium chloride (KCl) hypotonic solution, followed by a 20 min incubation at 37°C to swell the cells.

Cells were pre-fixed by adding 10% volume of freshly prepared Carnoy’s fixative (3:1 methanol:acetic acid, v/v), then washed twice with ice-cold Carnoy’s fixative and stored overnight at −20°C. The following day, the cell suspension was dropped from a height of ∼50 cm onto ice-cold glass slides held at a 45° angle. Slides were matured by incubation at 65°C for 1 h, followed by washing in 2× saline-sodium citrate (SSC) buffer. Chromosomes were stained with 1 μg/ml Hoechst 33 258 (Sigma–Aldrich) for visualization.

### Fluorescence *in situ*hybridization

Fixed metaphase spreads were washed in 2× SSC buffer and sequentially dehydrated ethanol series (70%, 85%, 100%) for 2 min each. Fluorescence *in situ* hybridization (FISH) probes (Empire Genomics; green CHR08-10-GR for chromosome 8 and orange MYC-20-OR for c-Myc) were applied to the slides, which were then sealed with rubber cement. Denaturation was performed on a hot plate at 75°C for 7 min, followed by hybridization for 16 h in a humidified chamber at 37°C.

After hybridization, coverslips were removed, and slides were washed in 0.3% Igepal CA-630 (Sigma–Aldrich) in 0.4 × SSC at 73°C for 2 min, followed by a wash in 0.1% Igepal CA-630 in 2× SSC at room temperature for 2 min. The slides were stained with 1 μg/ml Hoechst 33 258 (Sigma–Aldrich) and washed again in 2× SSC before being mounted with ProLong Diamond Antifade Mountant (Thermo Fisher Scientific) and covered with a coverslip.

FISH images were captured using a Zeiss LSM780 confocal microscope with a 63× objective. Image analysis was performed using Fiji (ImageJ-based software) [32].

### Chromosome flow cytometry and sorting

Actively dividing COLO 320DM and COLO 320HSR cells were synchronized in mitosis by treating with 0.4 μM nocodazole (Sigma–Aldrich) for 13 h. For RPE-1 cells, 100 μl of Colcemid (Gibco KaryoMAX Colcemid Solution in PBS) was added to 30 ml of culture medium. Following mitotic arrest, cells were harvested by mitotic shake-off and collected by centrifugation at 400 × *g* for 5 min at room temperature. Cells were resuspended in the medium containing nocodazole and incubated with 0.1 μg/ml latrunculin B (Cambridge Bioscience, CAY10010631) at 37°C for 1 h.

Cells were pelleted by centrifugation (400 × *g*, 5 min) and resuspended in a hypotonic solution containing 75 mM KCl, 10 mM MgSO_4_, 0.2 mM spermine, and 0.5 mM spermidine, pH 8.0. The cell suspension was incubated at room temperature for 20 min, followed by centrifugation at 300 × *g* for 5 min. Cells were then resuspended in ice-cold PAB buffer [150 mM Tris–HCl, pH 8.0, 0.25% Triton X-100, 20 mM KCl, 0.5 mM spermine, 1 mM ethylenediaminetetraacetic acid (EDTA), 5 mM ethylene glycol tetraacetic acid (EGTA)]. Chromosomes were released by gently flicking the tube, and the quality of chromosome release was checked by mixing 10 μl of sample with 1 μl of propidium iodide (PI) and visualizing under a microscope.

The isolated chromosomes were stained with a combination of 5 μg/ml 4',6-diamidino-2-phenylindole (DAPI), 50 μg/ml Chromomycin A3, and 10 mM MgSO_4_. The staining reaction was carried out in a low volume (300 μl) for 1 h at room temperature, followed by dilution with 2–3 ml of PAB buffer. Samples were then incubated for an additional 30 min.

Chromosomes were analysed and sorted using a BD FACSAria Fusion Flow Cytometer with a 70- or 100-μm nozzle, set at a flow rate of <25 000 events/s. The 585-nm filter was used to detect Chromomycin A3 and 450 nm filter was used to detect DAPI. Sorted chromosomes were maintained in PBS containing 1-mM EDTA for further analysis.

### Whole-genome sequencing and ecDNA assembly

Genomic DNA was extracted from cultured cells using the Monarch Genomic DNA Purification Kit (New England Biolabs) according to the manufacturer’s protocol. DNA libraries were prepared using the NEBNext^®^ Ultra™ II FS DNA Library Prep Kit for Illumina (New England Biolabs), following the manufacturer’s instructions. Library quantification and dilution were performed using the Qubit double-stranded DNA High Sensitivity Quantification Assay (Thermo Fisher Scientific). Sequencing was carried out on an Illumina NextSeq 2000 platform.

The resulting FASTQ files were aligned to the human reference genome (hg38) using BWA-MEM. AmpliconArchitect (AA; https://github.com/virajbdeshpande/AmpliconArchitect [33]) was employed to detect genomic amplifications. The output from AA was integrated with optical genome mapping data to facilitate the assembly of ecDNA structures using the Amplicon Classifier tool. Optical genome mapping was performed by the Wellcome Sanger Institute using the Saphyr^®^ instrument (Bionano Genomics). Cell pellets were prepared following standard Bionano protocols. DNA was labelled by DLE-1 and *de novo* assembled using the Bionano Access software.

### Repli-seq analysis of DNA replication timing

Repli-seq was performed as previously described with modifications [30, 34]. Briefly, actively proliferating cells were cultured in T175 flasks under standard conditions and pulse-labelled with 100-μM BrdU (Sigma–Aldrich) for 30 min at 37°C. After labelling, cells were washed twice with ice-cold PBS and fixed by adding 75% (v/v) ice-cold ethanol dropwise while vortexing gently. Cells were stored at −20°C for at least 16 h.

For FACS, fixed cells were washed with 1% (v/v) FBS in PBS and stained with a solution of PI (50 μg/ml, Sigma–Aldrich) and RNase A (20 μg/ml, Sigma–Aldrich) in PBS/1% FBS. After 30 min of incubation at room temperature in the dark, cells were filtered through a 37-μm nylon mesh and sorted by flow cytometry (BD FACSAria II) into five fractions representing different stages of S phases based on DNA content.

Genomic DNA was extracted from sorted cells using the Zymo Quick-DNA Microprep Kit (Zymo Research) following the manufacturer’s instructions. DNA was fragmented to an average size of 200 bp using a Covaris M220 ultrasonicator set to 75 W peak incident power, 10% duty cycle, 200 cycles per burst, for 260 s. Fragment size was verified using an Agilent Bioanalyzer 2100 with a high-sensitivity DNA chip.

Next, libraries were prepared using the NEBNext Ultra DNA Library Prep Kit for Illumina (NEB), according to the manufacturer’s instructions. BrdU-labeled DNA was immunoprecipitated by first denaturing the DNA at 95°C for 5 min, followed by incubation with an anti-BrdU antibody (BD Biosciences). Immunoprecipitated complexes were recovered by incubation with rabbit anti-mouse IgG and centrifugation. After washing, the samples were digested overnight with proteinase K (0.25 mg/ml) at 56°C. DNA was purified using the DNA Clean & Concentrator-5 kit (Zymo Research). The purified DNA was amplified, indexed, and prepared for sequencing using NEBNext Ultra II DNA Library Prep Kit for Illumina. Sequencing was performed on an Illumina NextSeq 2000 platform.

Sequencing reads were aligned to the human reference genome (GRCh38) using BWA-MEM. The aligned data were indexed, and polymerase chain reaction (PCR) duplicates and reads with multiple alignments were removed using SAMtools [35]. The resulting BAM files were normalized to reads per kilobase per million (RPKM) using bamCoverage. The normalized data were visualized in the Integrative Genomics Viewer (IGV) [36]. Repli-seq data for the colorectal cancer cell line HCT116 was taken from GSE137764 [29].

The statistical analysis of the synchronicity of the replication was achieved using Rao’s quadratic entropy (RQE) estimate. We consider bins of length 10kB, to which reads are mapped and normalized between five fractions encompassing the S phase, giving a categorical distribution over five classes. As a measure of disorder, we use RQE [37, 38] with a circular metric, which is more suitable for modelling cycling cells than alternatives not employing the temporal order of the categories, such as Shannon’s entropy [39]. The amplified fragment of length 1.6 Mb hence contains 160 bins, each associated with a single RQE estimate. We average this value over all bins, to associate a single estimate σ to each 1.6 Mb genomic fragment.

To assess the degree of extremity of the observed value, we calculate the empirical distribution of RQE estimate over fragments of matched length chosen uniformly along the whole genome, with the exception of fragments of length 5 Mb from each end to avoid sequencing artifacts. To avoid correlations between closely located fragments we ensure 10Mb spaces between subsequent fragments. This yields N = 273 fragments distributed along 22 chromosomes. After removing fragments containing bins without reads, we use N = 223 fragments of 1.6 Mb to estimate the empirical distribution. We compared the empirical distributions of RQE between the three cell lines using two-sample Kolmogorov–Smirnov test. All three comparisons returned highly significant *P*-values (<10^−19^ for HSR – DM; <10^−8^ for RPE-1 – HSR; 0.003 for RPE-1 – DM), suggesting minor experiment-specific differences. However, qualitatively the distributions look similar (see Fig. 2B). To assess the extremity of the RQE value associated with the *MYC* fragment, we calculated the tail probabilities *P* = P(X ≥ X_c-_*_MYC_*). We obtain p_DM < 0.01 and p_HSR < 0.01, with p_RPE-1 = 0.23. Our results therefore suggest that the replication of the amplified fragment in COLO 320DM and COLO 320HSR proceeds in a less ordered fashion than for the large majority of the matching fragments in these cell lines. This conclusion remains valid when the ecDNA interval, and corresponding fragment size in the RQE analysis, is taken as ∼4.3 Mb, the maximum reported size of the COLO 320DM ecDNA [7, 8, 40].

### Reverse transcriptase-polymerase chain reaction

Quantitative PCR (qPCR) was employed to estimate copy number variations of ecDNA by targeting the c-*MYC* gene (TaqMan™ Copy Number Assay, Assay ID: Hs00292858_cn). The human RNase P gene (TaqMan™ Copy Number Reference Assay, RNase P; Applied Biosystems, Cat. No. 4403328) was used as the reference.

Reactions were prepared using TaqPath™ ProAmp™ Master Mix (Thermo Fisher Scientific, Cat. No. A30865) according to the manufacturer’s instructions. Each 20 μl reaction consisted of 10 μl of 2 × TaqPath™ ProAmp™ Master Mix, 1 μl of 20 × TaqMan™ Copy Number Assay, 1 μl of 20 × TaqMan™ Copy Number Reference Assay (RNase P), and 2 μl of genomic DNA (1.6 ng). Amplification was conducted on an Viia7 (ThermoFisher) cycler under the following thermal cycling conditions: initial denaturation at 95°C for 10 min, followed by 40 cycles of 95°C for 15 s and 60°C for 60 s.

Relative copy number was calculated using the comparative Ct (ΔΔCt) method, normalizing target Ct values to the reference RNase P gene and comparing them to a TK-6 sample with a known copy number. All reactions were performed in technical quadruplicate across three biological replicates. Validation of qPCR detection sensitivity across a wide range (0–1000 copies of *c-Myc* plasmid DNA) was performed and visualized in Supplementary Fig. S9.

### Replication dynamics and origin analysis with DNAscent

Replication velocity and origins were analysed using DNAscent. Actively dividing cells in COLO 320DM-HU sample were optionally treated with 50 μM HU for 24 h to induce replication stress before being pulse-labelled with 50 μM EdU for 6 min, washed twice with warm PBS, and then treated with 50 μM BrdU for 6 min. HU was maintained in the medium during EdU and BrdU labelling. Cells were washed again with warm PBS and incubated with 100 μM thymidine for 1 h. After a final PBS wash, fresh complete medium was added. Note that for the experiments to examine HU-induced ecDNA loss, HU was maintained continually for 25 days at either 25 μM or 50 μM, by replenishing at each change of medium.

For PromethION (ONT) sequencing, cells were harvested 1 h after thymidine treatment, washed with ice-cold PBS containing 1% BSA, and flash-frozen in liquid nitrogen. Approximately 6 million cells were used for each experiment. DNA libraries were prepared for ultra-long sequencing using the Ultra-Long DNA Sequencing Kit (SQK-ULK1, ONT) and sequenced on a Nanopore PromethION platform. For one of the untreated DM samples (DM_untreated_rep2) adaptive sampling targeting chromosome 8 was performed (Supplementary Table S1). An initial attempt at enriching circles with pulse field gel electrophoresis resulted in a low N_50_ and no enrichment (Supplementary Table S1). N50 is calculated for reads passing the DNAscent quality criteria (minimum alignment length of 20 kb and minimum mapping quality of 20). Then, all reads are sorted by length in descending order, and the cumulative sum over all read lengths is calculated. To determine N50, the read where the cumulative sum up to that read is equal or greater than half of the overall cumulative sum, is determined, and this read’s length is the N50. An overview of all datasets is provided in Supplementary Table S1.

Alternatively, 30 min after cell labelling with BrdU and EdU, cells were arrested in mitosis by overnight treatment with 0.4 μM nocodazole. Following ecDNA release and FACS sorting (as described above), ecDNA was concentrated by centrifugation at 24 000 × *g* for 1 h at 4°C. DNA libraries were prepared using the Ligation Sequencing Kit (SQK-LSK110) and sequenced on a ONT MinION platform, following the manufacturer’s instructions.

Base calling of the raw sequencing files from ONT PromethION or MinION platform (R9.4.1 Nanopore, fast5 files available on ENA under accession PRJEB83636) was performed using GUPPY (version 5.0.16) with the configuration for R9-sequenced DNA (dna_r9.4.1_450bps_fast.cfg) and the output reads (fastq format) that passed and failed the base calling quality metrics were used for subsequent processing. Alignment of the reads to the hg38 reference genome (release GRCh38.p14) or our custom ecDNA reference map (see above) was performed with minimap2 (version 2.24). The output from minimap2 (sam format) was then converted to bam format and sorted and indexed using samtools (version 1.14). Next, DNAscent (version 3.1.2) subprogrammes index, detect, forkSense, and bedgraph (visualization) were run and replication forks and origins were visualized using a genome browser (IGV, version 2.15.1).

### Calculation of replication fork speed and outlier removal

The DNAscent forkSense output (bed file format, available on ENA under accession PRJEB83636) was used for custom downstream processing using Python (version 3.8.2), starting with calculation of fork speed. Replication fork speed and stall scores from DNAscent were obtained as previously described [41]. We only include replication forks where both the fork speed and stall score could be calculated for downstream analyses i.e. fork tracks were not near the end of the read. Reads that mapped to chromosome Y were removed as the cell line originates from the tumour of a female patient [42]. For all fork speed figures in the main body, we apply outlier filtering based on the interquartile range (IQR). Outliers were removed based on the IQR. The first (Q1, 25%) and third quartiles (Q3, 75%) were calculated, and the IQR was defined as the difference between Q3 and Q1. Outliers were identified as data points lying below the lower bound (Q1 − 1.5 × IQR) or above the upper bound (Q3 + 1.5 × IQR) and were excluded from further analysis. For the Supplementary histograms of fork speed, no IQR filtering was applied to present the raw data (Supplementary Fig. S6). A replicate comparison for the PromethION runs is provided in Supplementary Fig. S7. Data cleaning, outlier removal, statistics, and visualizations were performed using custom Python (version 3.11.5) scripts, except for circular plots which were created in R (see below).

### FACS enrichment analysis

To assess the enrichment factor achieved through FACS sorting, we compared the ratio of reads mapped to ecDNA and to the human reference genome, focusing only on reads that passed the DNAscent minimum quality criteria (minimum alignment length of 20 kb and minimum mapping quality of 20).

### Visualization of ecDNA maps with origin densities, fork speeds, and stall scores

Circular ecDNA visualizations were generated using the *circlize* package (version 0.4.16) [43] in R (version 4.3.3) using the IQR filtered replication forks as input for fork speed and stall scores and DNAscent forkSense origins for origin of replication analyses. In brief, a custom backbone was created using the AmpliconArchitect [33] generated ecDNA reference map. For origin of replication visualizations, origins from all biological replicates of COLO 320DM (‘DM’, four replicates), COLO 320HSR (‘HSR’, four replicates), or DM HU treated (‘DM-HU’, three replicates) were pooled. Origins were grouped into 50-kb segments and normalized by the number of reads in each segment to account for varying read depth within and across samples. The number of reads in each segment was obtained from the aligned bam file and only reads that passed the DNAscent quality criteria (minimum alignment length of 20 kb and minimum mapping quality of 20) were included for the read depth assessment. Aligned read length and mapping quality were extracted from the bam file using the *pysam* package (https://github.com/pysam-developers/pysam) in Python. For replication fork speed and stall scores, replication forks for each group (DM, HSR, DM-HU) from all replicates were pooled and grouped into 20 kb segments. Fork speed and stall score averages across all forks in each segment were calculated for visualization. G-quadruplex scores for the ecDNA reference map were obtained using G4Hunter [44] with a window size of 25 and a threshold of 1.5.

## Results

### Molecular characterization and isolation of ecDNA

**Figure 1.**
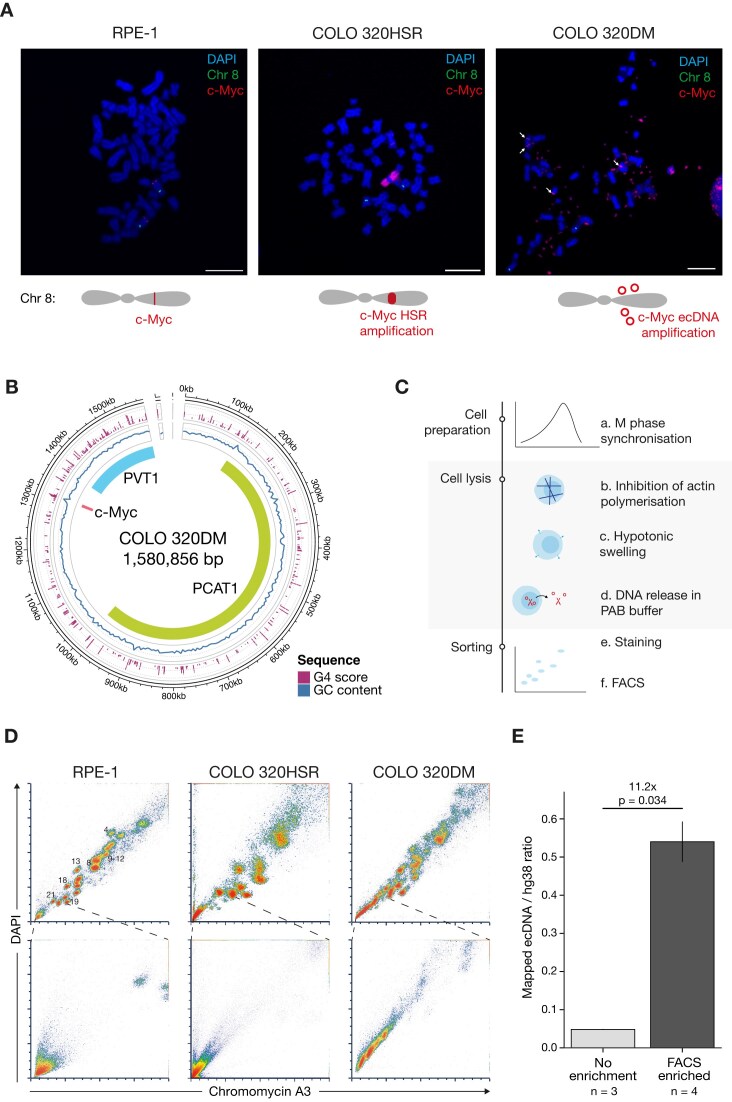
Characterization of ecDNA in COLO 320DM and its isolation by FACS. (**A**) Representative DNA FISH image of c-*MYC* localization in RPE-1, COLO 320HSR, and COLO 320DM. ecDNA or the HSR were labelled by FISH with a c-*MYC* probe. Slides were stained with DAPI, 5-fluorescein (centromeric region of chromosome 8) and 5-TAMRA (*c-MYC*). Although a previous study had reported that the FISH for c-*MYC* is found only on ecDNAs in COLO 320DM [49], we observed some *c-MYC* signal within chromosomes at a frequency of < ∼0.04 (Fig. 1A, right panel, white arrows) which likely represent ecDNA reintegration events. Scale bar, 10 μm. The cartoons provide a graphic representation of the *MYC* amplification in COLO 320HSR and DM. (**B**) Composite graph depicting the ecDNA structure in COLO 320DM generated using short reads and optical genome mapping with Amplicon Reconstructor. G4 calls from G4Hunter [44] above threshold 1.5 (purple dots); grey dotted lines are shown at G4Hunter scores 2.0 and 3.0); GC content (blue line); grey dotted line shown at 50%. (**C**) Experimental workflow of the FACS-based protocol for ecDNA isolation involving cell preparation, DNA release and FACS sorting. (**D**) Flow cytometry plots of chromosomes (top) and the region containing ecDNA and debris (bottom) from RPE-1 (left), COLO 320HSR (centre), and COLO 320DM (right) cell lines. The scales represent linear mean fluorescence intensity but do not reflect detector voltage gains. Chromosome detection was performed using a detector gain of 622 V (DAPI) and 651 V (Chromomycin A3) for COLO 320DM; 622 V (DAPI) and 604V (Chromomycin A3) for RPE-1; and 668V (DAPI) and 713 V (Chromomycin A3) for COLO 320HSR. ecDNA detection was set using a gain of 750 V (DAPI) and 750V (Chromomycin A3) for COLO 320DM; 750 V (DAPI) and 750 V (Chromomycin A3) for RPE-1; and 801 V (DAPI) and 875 V (Chromomycin A3) for COLO 320HSR. (**E**) Ratio of ONT long read counts of sequencing reads aligned to the ecDNA region. Left: Whole genome results excluding adaptive sampling sequencing, right: following FACS-based ecDNA purification. n indicates number of biological replicates. Error bars represent standard deviation.

To investigate ecDNA replication, we used the colorectal cancer cell line COLO 320DM [42], which carries an amplification of the c-*MYC* locus on multiple ecDNAs. A cell line (COLO 320HSR) derived from the same patient tumour provides an interesting comparison in that it harbours linear amplifications of a region of chromosome 8 containing c-*MYC* on another chromosome [42]. We also used an unrelated, immortalized, but untransformed cell line RPE-1 as a control [45] (Fig. 1A).

Previous studies have reported substantial variation in the size of ecDNA molecules in the COLO 320DM cell line of between ∼1.1 Mb and over 4.3 Mb, with some of the larger amplicons containing multiple copies of the c-Myc oncogene [7–10, 40, 46]. This variability likely reflects natural evolution of the ecDNA during cell passage and thus cell line batch variation. To minimize variability in the present study, we used low-passage COLO 320DM obtained from ATCC. We characterized the ecDNA structure in our isolate of COLO 320DM, using AmpliconArchitect [33] and AmpliconReconstructor [47] algorithms that integrate optical genome mapping with whole-genome short-read sequencing (Fig. 1B). The results suggested the presence of a heterogenous pool of ecDNAs with all the dominant species mapping consistently to a 1.6 Mb region at 8q24 around the c-Myc locus. Therefore, we used this map as a reference for downstream analyses and focussed our analyses primarily on the amplified sequence and replication dynamics of this interval, rather than attempting to map replication to precise structure of different ecDNA species.

Existing methods for ecDNA isolation pose significant challenges, requiring ecDNA cleavage or chromatin digestion, resulting in the extraction of limited data with high levels of noise [13, 48]. To overcome these limitations, we refined established protocols for mitotic chromosome karyotyping by employing FACS at the limits of its capabilities to detect and isolate ecDNA. In addition to previously tested approaches, we introduced latrunculin B treatment to reduce the internal structural integrity of the cells by disrupting the actin cytoskeleton, which allowed us to omit the vortexing step. As a result, mitotic DNA, including both chromosomes and ecDNA, was released with minimal shearing, generating less debris and improving resolution, allowing for clearer visualization of ecDNA that would otherwise be obscured by larger, chromosome-derived, DNA fragments (Fig. 1C). This method is efficient in visualizing and sorting intact chromosomes in chromosomally stable cell lines such as RPE-1 (Fig. 1D), as well as in aneuploid cancer cell lines like COLO 320DM and COLO 320HSR (Fig. 1D).

As anticipated, the substantial chromosomal heterogeneity observed in metaphase spreads of COLO 320DM led to less distinct chromosome profiles (Fig. 1D). However, by increasing the voltage during sorting, we were able to detect low-molecular-weight DNA populations exclusive to ecDNA-positive cell lines, which were absent in ecDNA-negative COLO 320HSR and RPE-1 (Fig. 1D), suggesting that these populations represent ecDNA rather than simple debris. Subsequent sequencing and copy number analysis revealed a 11–40-fold enrichment of ecDNA following FACS sorting compared to sequencing without prior isolation, further confirming that the isolated DNA molecules were ecDNA (Fig. 1E).

### Replication timing of ecDNA

To examine the timing of ecDNA replication, we employed the Repli-Seq technique, commonly used to assess replication timing in chromosomal DNA [29]. We used five FACS gates across the cell cycle which provided the temporal resolution necessary not only to distinguish between early- and late-replicating regions, but also to assess whether ecDNA replication is synchronous within the population and whether it reflects the normal timing of the region when in a chromosomal context.

**Figure 2.**
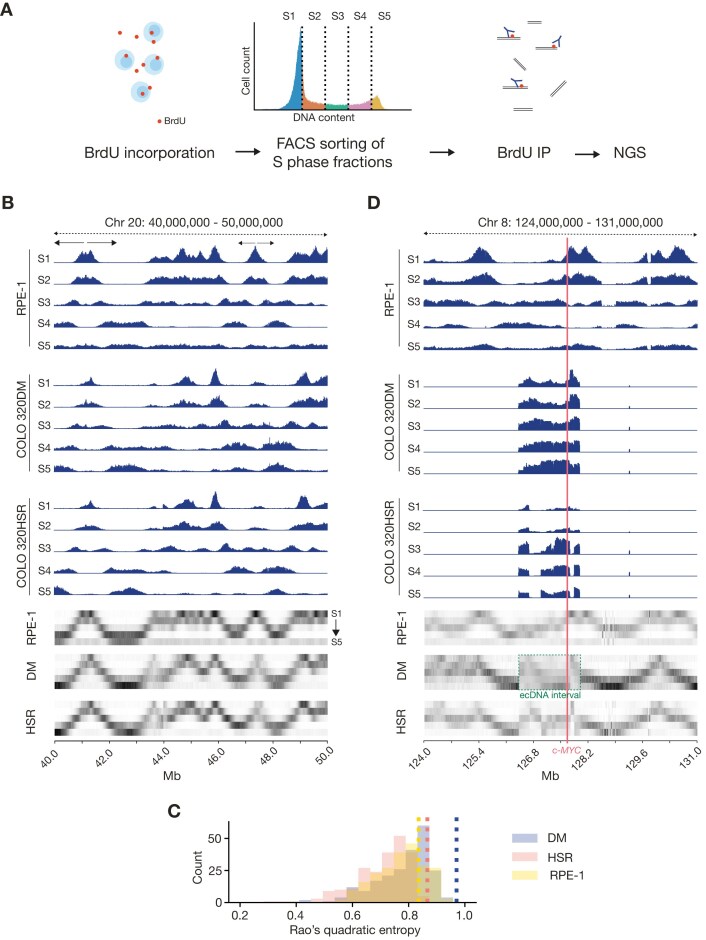
ecDNA replicates throughout S phase in COLO 320DM. (**A**) Graphical representation of Repli-seq workflow. (**B**) Replication timing analysis of a 10 Mb region on chromosome 20 (40 000 000–50 000 000) in RPE-1, COLO 320DM, and COLO 320HSR. Coverage shown as RPKM mapped reads, group normalized to the highest peak in the visualized region between samples from the same cell line. Lower panels: Visualization of the proportion of reads in each S-phase bin as a function of genome location. (**C**) Rao entropy of replication timing. Replication timing was analysed using 1.6 Mb genomic bins (matching the ecDNA size in COLO 320DM), spaced 10 Mb apart. The Rao entropy for each bin was calculated for all three cell lines. Dashed lines indicate the Rao entropy of c-*MYC* gene in each respective cell line. (**D**) Replication timing analysis of the region around ecDNA locus on chromosome 8 in RPE-1, COLO 320DM (DM), and COLO 320HSR (HSR). Coverage shown as RPKM, group normalized to the highest peak in the visualized region between samples from the same cell line. Lower panels: Visualization of the proportion of reads in each S-phase bin as a function of genome location. ecDNA region highlighted with green dashed box. c-*MYC* labelled in red. Note that the ecDNA regions in COLO 320DM and COLO 320HSR are shown aligned contiguously with adjacent sequence on chromosome 8 although the integration of the sequence in COLO 320HSR is not on chromosome 8 (Fig. 1A).

We performed Repli-Seq in COLO 320DM, COLO 320HSR, and RPE-1. Each cell line was labelled with BrdU for 30 min, fixed in Carnoy’s fixative, stained with PI, and subjected to sorting into five separate fractions before deep sequencing of each fraction (Fig. 2A).

To depict replication timing of ecDNA, we plotted the fraction of reads in windows of 10 kb across the genome for each S-phase bin (Fig. 2B). Examination of a representative genomic region on chromosome 20 in the three cells lines suggested that they exhibit qualitatively similar replication timing profiles, with minor experiment-specific differences (Fig. 2B; see the ‘Materials and methods’ section). We also compared previously published replication timing data [29] for the same region on chromosome 20 in another colorectal cancer cell line, HCT116, which showed a similar replication pattern (Supplementary Fig. S1).

To test the conservation of this replication timing pattern across the genome, we measured replication synchronicity using the normalized mean RQE [37, 38], which is an information theory-based measure of distribution diversity, that considers the temporal ordering of the S-phase fractions (see the ‘Materials and methods’ section). In this analysis, a fragment replicating fully in a single fraction of S phase has a RQE of zero, while a fragment replicating uniformly across all five subphases achieves RQE of exactly 1. We plotted RQE of 1.6 Mb fragments (chosen to match the size of the ecDNA in COLO320DM) across the whole genome (Fig. 2C). While differences in the distribution of synchronicity of replication timing are observed across the genome in the three lines, as would be expected for transformed cells [50, 51], there is significant overlap in the distributions. Notably, none of the 1.5-Mb fragments sampled was found to replicate as asynchronously as ecDNA (Fig. 2B).

However, replication of the ecDNA region in COLO 320DM appears considerably less synchronous than in the RPE-1 control with ongoing replication observed throughout the circle in the entire S phase (Fig. 2D). This observation is supported by an RQE of 0.96, close to the maximum possible value of 1 and represents an outlier in the distribution of replication timing synchronicities in all three cell lines (Fig. 2C). The same region in COLO 320HSR, in which the circle DNA is chromosomally integrated as an array, exhibits broadly late replication timing throughout, in contrast to the wave of replication that broadly passes left to right through the region in RPE-1 cells as S-phase progresses (Fig. 2D), but the overall synchronicity of the region is similar to that seen in RPE-1. To further validate this observation, we compared our data to previously published replication timing data from another colorectal cancer cell line, HCT116 [29]. We observed a replication timing pattern more similar to RPE1, with c-Myc replicating in early-to-mid S phase in both datasets (Supplementary Fig. S1). Thus, replication on the ecDNA in COLO 320DM is significantly less ordered in terms of timing than the genome as a whole.

We next investigated replication fork velocity and origin distribution in the COLO320DM ecDNA.

### Replication origin distribution on ecDNA

**Figure 3.**
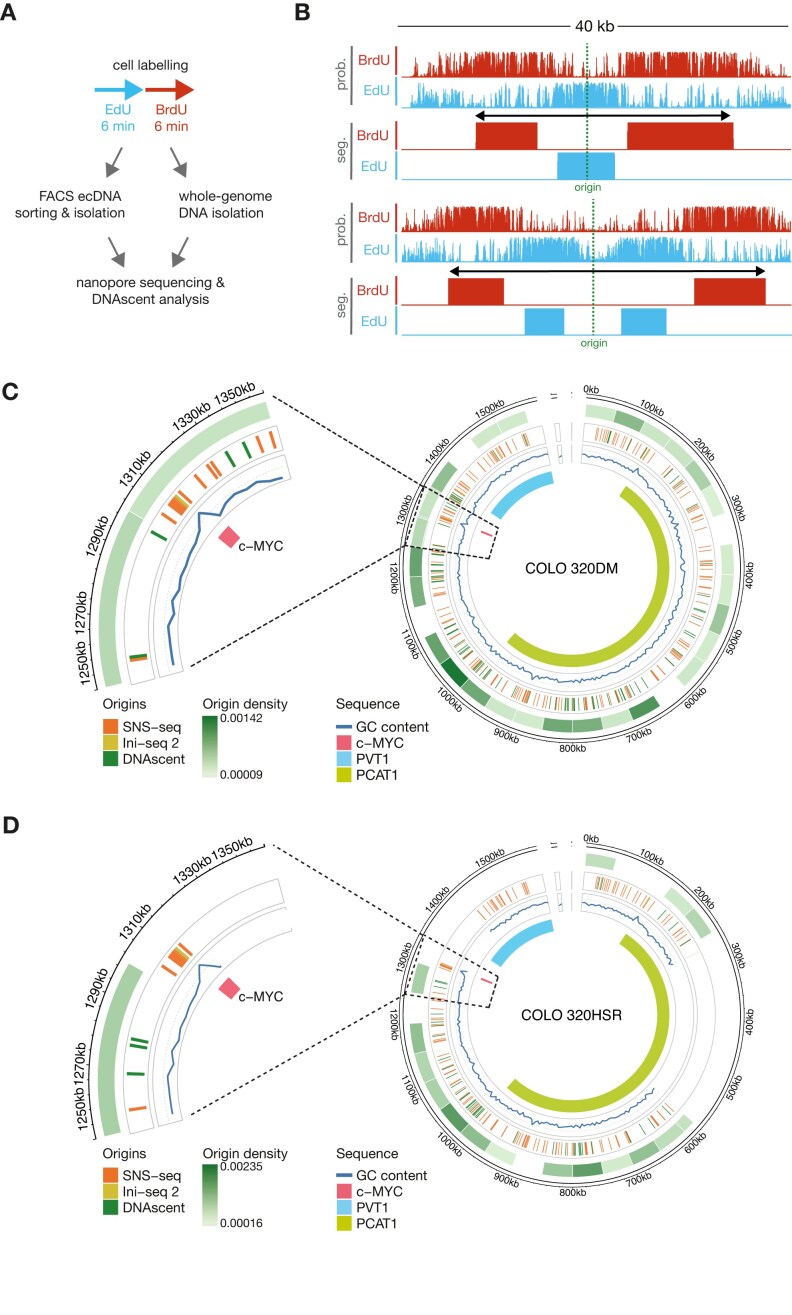
Location of origins of replication. (**A**) Schematic of base analogue pulsing protocol followed by either FACS sorting or whole genome DNA extraction for subsequent ultra-long Nanopore sequencing and DNAscent analysis. (**B**) Representative raw base analogue incorporation probabilities and DNAscent segmentation of origins of replication each represented by four tracks (upper tracks: Raw BrdU and EdU probabilities (prob.) at thymidine positions; lower tracks BrdU and EdU segmentation (seg.) derived from the raw probabilities). Both origins map to ecDNA in the untreated COLO 320DM cell line. (Coordinates on ecDNA map to chromosome chr8_126 425 747–127 997 820 bp. The top origin track maps within the reconstructed ecDNA between 793 008 and 801 870 bp; the lower example to 470 941–470 941 bp.) Raw DNAscent probabilities for EdU or BrdU are shown on a scale from 0 to 1, segmentation is binary. (**C**, **D**) Distribution of origins in panel (C) COLO 320DM and panel (D) Colo 320HSR on the ecDNA reference map (low density = light green shading to high density = dark green shading). Orange: SNS-seq origin locations [53]; yellow: Ini-seq 2 origins [54]; green: DNAscent origin locations; blue line: GC content. Zoom in shows the region around c-*MYC* (1250–1350 kb).

Given the less synchronized replication observed in ecDNA (Fig. 2), we explored whether ecDNA employs a different number and/or position of origins compared with the corresponding genomic sequence in a non-ecDNA context. We employed DNAscent [31] to identify replication forks and origins of replication in the sequences containing c-*MYC* amplified either on ecDNA or in the HSR. In brief, cells were incubated with two nucleoside analogues EdU and BrdU for 6 min each, which were incorporated into newly synthesized DNA during replication. The isolated DNA was then subjected to ultra-long ONT sequencing (Fig. 3A). The characteristic disruptions in electrical current caused by EdU and BrdU as the labelled DNA passes through the sequencing pores allows the probability of their presence to be calculated by the DNAscent model on top of the canonical DNA sequence. The distribution of EdU and BrdU along the individual DNA molecules is indicated as a probability (Fig. 3B). Applying density-based segmentation to these probabilities creates replication tracts that identify fork direction, velocity, stalling, and replication origins [31].

We initially employed DNAscent on FACS-isolated ecDNA. While this allowed identification of replication forks and replication origins in ecDNA (Supplementary Fig. S2), the throughput was not satisfactory and the need to concentrate the circle DNA from the relatively large volumes generated by FACS sorting led to DNA breakage and relatively short reads in the ONT sequencing runs (Supplementary Table S1). We therefore moved to using whole-genome sequencing using the ONT PromethION instrument.

Using PromethION sequencing on unselected DNA we were able to achieve an average N50 of 91.1 ± 16.7 kb (Supplementary Table S1). From this data, we identified 1312 reads with origin of replication calls in COLO 320DM and 1115 reads with origin calls in COLO 320HSR DNA. Of these, 81 and 43 respectively mapped to the 1.6 Mb interval covered by the ecDNA (Supplementary Table S1). The sites to which origins were mapped in the ecDNA interval exhibited no significant increase in GC content or G4 prevalence over randomly selected regions from the same interval (Supplementary Figs S3 and S4). We examined whether the distribution of origins is conserved between these amplification types, given differences in replication timing (Fig. 2D). There is not a significantly higher origin density in the ecDNA of COLO 320DM compared with the equivalent interval in COLO 320HSR, consistent with the genome-wide analysis (Supplementary Fig. S5). Further specific analysis of the region around c-*MYC*, which contains known sites of replication initiation [52], revealed origins near the c-*MYC* and *PVT1* loci in both cell lines. However, these did not align with the origins within c-*MYC* gene observed in other systems (Fig. 3C and D), highlighting potential differences in the replication patterns of the amplified c-*MYC* locus in COLO320 cell lines [53, 54] and consistent with the disorganized replication timing of this region in the COLO320 DM ecDNA (Fig. 2). Origin distribution across the ecDNA differed significantly from a uniform distribution based on origin counts in 50kb segments for all 3 conditions as determined by a one-sample Kolmogorov–Smirnov test (COLO 320DM: KS statistic = 0.91, *P*-value = 1.9e-34; COLO 320HSR KS statistic = 0.76, *P*-value = 7.6e-9; COLO320 DM HU-treated KS statistic = 0.92, *P*-value = 5.9e-36).

### Replication forks on ecDNA progress more slowly than on most chromosomal regions

**Figure 4.**
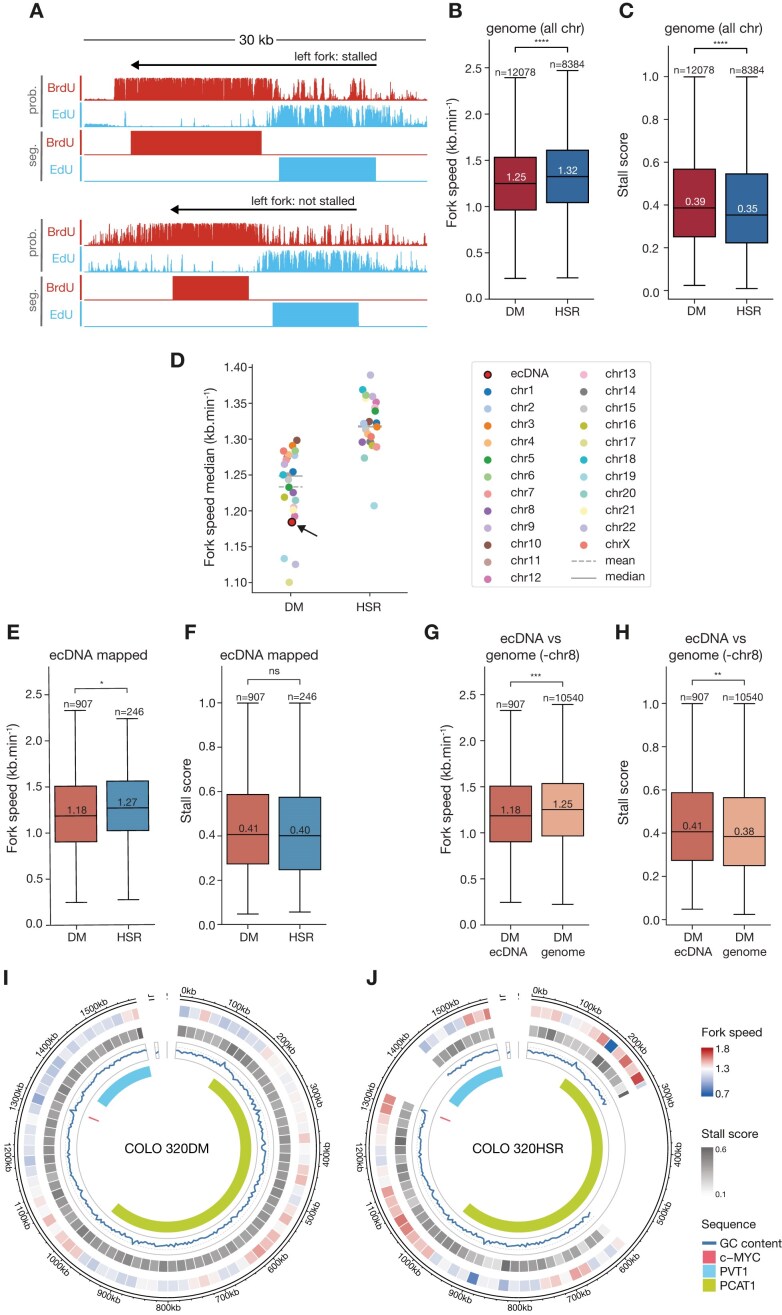
Replication dynamics on ecDNA. (**A**) Representative DNAscent tracks for leftward-moving replication forks [BrdU probability (prob.) and segmentation (seg): red; EdU: blue]. The top example shows a stalled fork, characterized by a sharp drop-off in BrdU incorporation probability at the fork tip (upper tracks; stall score of 0.9991, fork speed of 1.79 kb/min). The lower example tracks show a replication fork with no fork stalling (stall score of 0.2657, fork speed 1.36 kb/min) represented by a smooth decrease in BrdU probabilities at the fork tip. Both examples are from the Colo 320HSR cell line within the ecDNA region from chromosome 8. Raw DNAscent probabilities for EdU or BrdU are shown on a scale from 0 to 1, segmentation is binary. (**B**, **C**) Comparison of genome-wide fork speeds (**B**) and stall scores (**C**) in COLO 320DM (DM; red) and Colo 320HSR (HSR; blue). (**D**) Median fork speed per chromosome in COLO 320DM (DM) and COLO 320HSR (HSR) cell lines. For the COLO 320DM cell line, the median fork speed on the ecDNA is shown as a red dot with black outline. (**E**, **F**) Comparison of fork speed (**E**) and stall scores (**F**) of forks mapped to the ecDNA interval in COLO 320DM (circular, light red) and COLO 320HSR (chromosomally reintegrated, light blue). (**G**, **H**) Comparison of fork speed (**G**) and stall scores (**H**) within COLO 320DM cell line between forks mapped to the ecDNA interval (dark orange) and forks mapped to chromosomes (excluding chromosome 8 from which the ecDNA originates, light orange). (**I**, **J**) Visualization of replication fork speeds (**I**) and stall scores (**J**) averaged across 20-kb segments of the ecDNA interval in COLO 320DM and (**J**) COLO 320HSR cell line. Outer track, fork speeds; second track, stall score; third track, GC content (blue line); inner track, genes. Fork speeds and stall scores representation uses the same range in panels (I) and (J) with the most extreme values across both cell lines determining the minimum and maximum colour shades. All *P*-values for boxplots with a fork speed are obtained from a two-sided Welch’s *t*-test with no assumption of equal variances and all *P*-values in boxplots for stall scores are obtained from a two-sided nonparametric Wilcoxon rank-sum test. Statistical significance: ns, not significant (*P* ≥.05), *P <.05, **P <.01, ***Pp <.001, ****P <.0001.

To further investigate the differences in replication dynamics between ecDNA and the chromosomes, we measured replication fork velocity and stalling in ecDNA-positive and ecDNA-negative cell lines. Using ONT sequencing and DNAscent, we detected individual nascent DNA fragments labelled with EdU and BrdU (Fig. 4A). By dividing the length of continuous stretches of labelled DNA by the pulse durations we calculated replication fork velocity—longer tracts indicate faster overall fork progression. Additionally, we examined the pattern of signal decay to assess fork stalling: an abrupt loss of signal corresponds to replication fork stalling or termination, while a gradual decline reflects normal fork progression as BrdU levels diminish over time due to the thymidine chase (Fig. 4A). This approach enabled us to directly measure and compare replication fork dynamics on ecDNA and chromosomal DNA, revealing statistically significant differences in fork velocity and stalling patterns [31].

Global fork velocity was reduced genome-wide in ecDNA-positive COLO 320DM compared to ecDNA-null COLO 320HSR (Fig. 4B). Moreover, ecDNA-positive cells displayed a higher frequency of fork stalling (Fig. 4C), though this increase in stalling did not account for the reduced fork velocity since forks selected for matched levels of stalling still exhibited reduced fork velocity in COLO 320DM (Supplementary Fig. S6). Consistent with these findings, replication forks on each chromosome in COLO 320HSR were progressing more rapidly than on its counterpart in COLO 320DM cells (Fig. 4D).

To further characterize ecDNA replication, we compared the replication dynamics of ecDNA with its corresponding amplified sequence on the HSR in COLO 320HSR cells. Replication of the ecDNA was ∼6.5% slower (1.27 versus 1.18 kb/min), although no significant difference in fork stalling rates was observed (Fig. 4E and F). Similarly, ecDNA replication in COLO 320DM was slower than chromosomal DNA replication (1.18 versus 1.25 kb/min; Fig. 4G and H), with a marginally higher stalling rate (0.41 versus 0.38).

We next examined the distribution of replication fork velocities across COLO 320DM ecDNA and the corresponding sequence in COLO 320HSR. Mean fork velocity, visualized in 20 kb bins, ranged from 0.7 to 1.8 kb/min, with most regions showing concordant replication velocities between ecDNA and HSR (Fig. 4I and J). However, regions such as the c-*MYC* locus exhibited stark differences in replication dynamics. While the c-*MYC* region was one of the fastest-replicating in the HSR, it was the slowest-replicating on ecDNA, indicating substantial differences in replication dynamics between ecDNA and chromosomal DNA (Fig. 4I and J). A similar pattern was observed for the stall rate (Fig. 4I and J), with ecDNA displaying elevated stalling in regions where HSR replication was most efficient. These findings suggest that ecDNA replication diverges significantly from chromosomal HSR replication, even in the regions with the same underlying sequence and, overall, consistent with replication on ecDNA being more intrinsically stressed [14].

### HU disrupts ecDNA replication, leading to its depletion from cells

**Figure 5.**
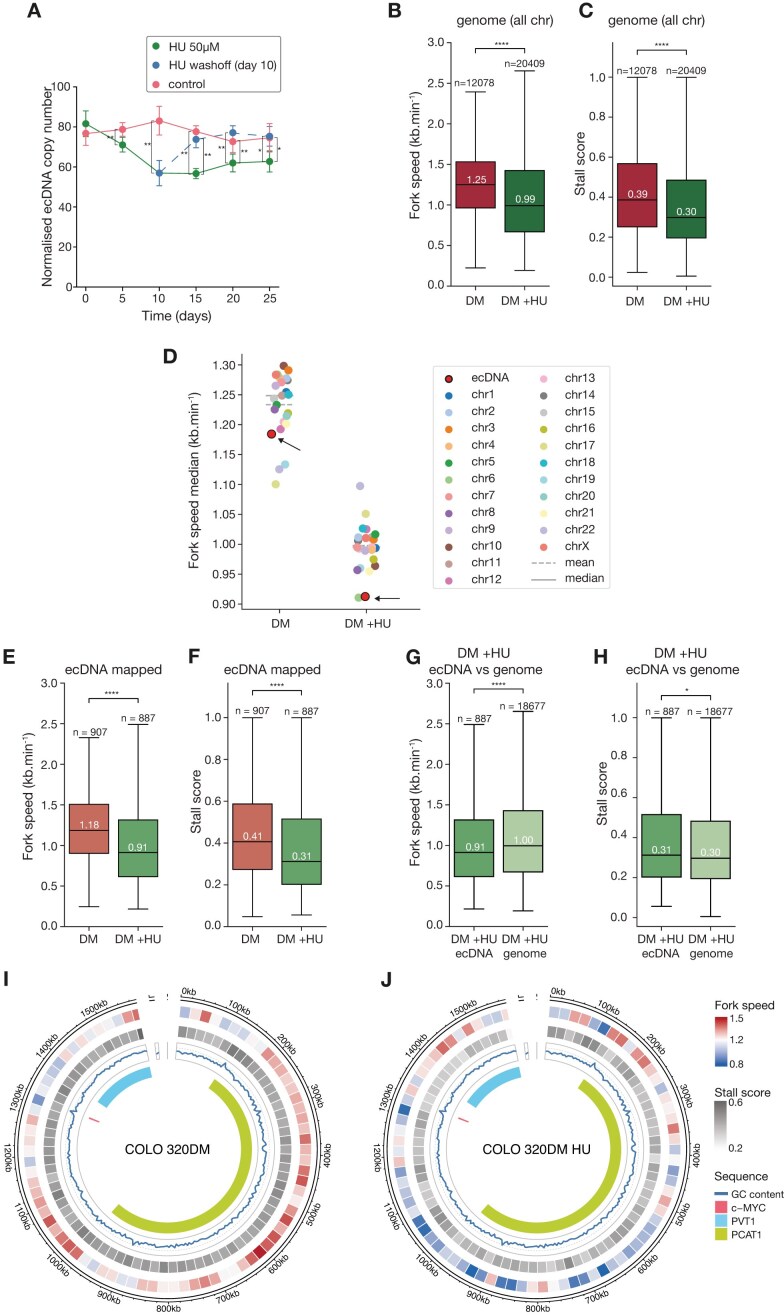
HU treatment slows down DNA replication on ecDNA. (**A**) Copy number variation, estimated by qPCR, in COLO 320DM following continuous exposure to 50 μM HU over 10 days (green), upon HU removal (blue), and without treatment (red). Pair-wise comparisons are assessed with a two-sided Wilcoxon rank-sum test. (**B**, **C**) Fork speeds (**B**) and stall scores (**C**) in untreated (red) and HU-treated (green) COLO 320DM cell lines. Replication dynamics was assessed after 24-h treatment with 50 μM HU. (**D**) Median fork speed per chromosome in untreated (DM) and HU-treated (DM_HU) COLO 320DM cell lines. The median fork speed on ecDNA is shown as a red dot with a black edge. (**E**, **F**) Comparison of fork speed (**E**) and stall scores (**F**) stall on ecDNA region mapped forks in untreated (medium red) and HU-treated (medium green) COLO 320DM cell line. (**G**, **H**) Comparison of fork speed (**G**) and stall scores (**H**) in forks mapped to ecDNA (medium green) and forks mapped to chromosomes (excluding chromosome 8), light green from HU-treated COLO 320DM cells. (**I**, **J**) Visualization of replication fork speeds and stall scores averaged across 20kb segments in the ecDNA interval of untreated (**I**) and HU-treated (**J**) COLO 320DM cells. Fork speeds lower than the average fork speed (light grey) are shaded in blue, higher in red and stall score averages are all low to moderate (light to medium grey). GC content is shown as a blue line and position of c-*MYC* and *PVT1* genes are highlighted by pink and blue blocks, respectively. Colouring of fork speeds and stall scores is the same in panels (I) and (J) with the most extreme values across both cell lines setting the minimum and maximum colour shades. All *P*-values for panel (A) are obtained from a Wilcoxon rank-sum test. All *P*-values for boxplots with a fork speed are obtained from a two-sided Welch’s *t*-test with no assumption of equal variances and all *P*-values in boxplots for stall scores are obtained from a two-sided nonparametric Wilcoxon rank-sum test. Statistical significance: ns, not significant (*P* ≥.05), *P <.05, **P <.01, ***P <.001, ****P <.0001.

Consistent with previous studies [25–28], HU treatment led to a marked reduction in ecDNA levels in COLO 320DM cells (Fig. 5A). This was reversed on withdrawing HU (Fig. 5A), suggesting strong selective pressure to maintain ecDNA in these cells. To further explore this phenomenon, we analysed ecDNA replication dynamics under HU treatment using DNAscent.

First, we investigated changes in origin distribution under HU treatment, as reduced fork velocity can trigger activation of dormant origins to compensate for slower replication [55, 56]. Upon HU treatment, we observed increased origin activation both genome-wide and on ecDNA (Supplementary Fig. S5), particularly around the c-*MYC* locus (Supplementary Fig. S3). This shows that dormant origins are activated both genome-wide and on ecDNA under replication stress.

HU treatment also resulted in a genome-wide reduction in fork velocity of 20.7% (Fig. 5B; 0.99 versus 1.25 kb/min), with every chromosome exhibiting decreased replication speed (Fig. 5D). Notably, DNAscent-detected fork stalling also decreased in HU treated cells (Fig. 5C). This suggests that the reduced velocity of DNA synthesis resulting from nucleotide depletion leads to fewer sharply cut-off tracts of DNA synthesis, which likely represent stalled forks [31], is perhaps counterintuitive. However, such resolution of stalls has previously not been accessible to techniques like DNA combing due to their inability to distinguish between gradual decline of the signal, and abrupt signal loss suggesting stalling [57]. The observed reduction in the stall score may reflect either the slower forks in HU less frequently encountering synthesis impediments, or the slower replication allows more opportunity to deal with problems on the template ‘on-the-fly’ allowing synthesis to continue. This reduction in fork velocity, observed both on ecDNA and genome-wide, may also lead to underreplication, which might be mitigated by the increased activation of dormant origins in response to HU-induced replication stress.

When comparing specifically ecDNA replication under HU treatment, the replication slowing effect was even more pronounced. Fork velocity on ecDNA dropped significantly by 22.9% (0.91 versus 1.18 kb/min), making it the slowest replicating fraction relative to all chromosomes (Fig. 5D and E). The stalling rate also decreased from 0.41 to 0.31 (Fig. 5F), indicating that stalled forks do not account for the overall reduction in replication speed. Even under HU treatment, ecDNA replication remained slower than chromosomal DNA (Fig. 5G; 0.91 versus 1 kb/min), with an even higher relative decrease in replication velocity (5.6% versus 9% reduction) (Supplementary Fig. S8). Notably, the stalling rate across both fractions dropped to nearly identical levels (0.31 versus 0.30) (Fig. 5H). These effects were observed in both ecDNA and chromosomal DNA but to different extents. Replication fork velocity decreased proportionally more on ecDNA, while the stalling rate was similarly reduced in both fractions (Supplementary Fig. S8), which may contribute to the loss of ecDNA from cells under HU treatment.

Further analysis of 20 kb fragments across ecDNA confirmed a significant reduction in both replication velocity and stalling (Fig. 5I and J). However, the distribution of fast- and slow-replicating regions shifted markedly. Regions surrounding c-*MYC* and *PVT1*, previously among the slowest replicating areas, became the fastest replicating upon HU treatment, even surpassing their pre-treatment speeds. This suggests that HU has its greatest impact on the fastest replicating regions, indicating a complex, region-specific influence on ecDNA replication. In contrast, the stalling rate across the other regions of ecDNA decreased more uniformly, suggesting a differential impact of HU on replication dynamics that may be driven by the variations in underlying sequence or local chromatin environment (Fig. 5I and J).

## Discussion

This study reveals that ecDNA replicates asynchronously throughout the S phase, contrasting with the well-defined replication timing of linear chromosomal DNA. While some of this asynchronicity likely reflects the independent nature of multiple copies of the ecDNA, there may also be a contribution from asynchrony of replication between copies of the amplified interval within ecDNA molecules, and to a lesser extent a contribution from subclones of cells in which some ecDNA has reintegrated into chromosomes. Nonetheless, the net effect is that the chromosomal replication timing of the amplified interval is lost. This asynchrony suggests that ecDNA lacks the strict temporal regulation of replication initiation found in chromosomal DNA, possibly due to its previously reported generally accessible euchromatic nature [9, 58].

Given the asynchronous replication on ecDNA, we compared origin usage with the corresponding chromosomal sequence. The different origin usage we observed on ecDNA could influence both replication dynamics and the potential for replication-transcription conflicts. A mildly reduced fork velocity and elevated stalling rates on ecDNA under normal conditions suggest that replication forks on ecDNA are indeed constitutively subject to some degree of replication stress. That said the precise cause or causes of slower unperturbed DNA replication on ecDNA remains to be fully understood, but is noteworthy that ecDNA is reported to exhibit a more open chromatin conformation than chromosomal DNA [9]. This may both increase the probability of replication initiation, as observed for the distribution of chromosomal replication origins [54], or conversely, impede it by generating high levels of replication-transcription conflicts [14, 59], which while not universally observed across all ecDNA-positive cell lines [15, 16], is an important feature of ecDNA in COLO 320DM. Following HU exposure, which will exacerbate replication stress by depleting nucleotide pools, slowing of ecDNA replication is accompanied by the activation of additional origins suggesting that the well-documented compensatory mechanism by which cryptic origins are fired to ensure complete replication despite slower fork progression [55, 56] is active on ecDNA.

Although previous work using DNA fibre analysis experiments has also reported overall slow replication slowing on ecDNA [14], consistent with our observations, DNAscent allows a distinction between slowing and complete stalling of DNA synthesis [41] allowing us to make the somewhat surprising observation that, in the presence of HU, while there is clear fork slowing there is also a reduction in the frequency with which forks stall. This suggests that slower replication forks are actually less prone to complete stalling of DNA synthesis. This observation contrasts with previous reports that HU treatment is linked to increased fork stalling [60]. This discrepancy may be the result of the fact that classical optical analysis of DNA synthesis in DNA fibres does not have the resolution to distinguish between slower replication and increased stalling.

We also hypothesize that the depletion of ecDNA under HU treatment is likely due to its vulnerability to replication stress and possibly the less efficient or accurate operation of mechanisms to rescue distressed forks, an idea that requires further investigation. This observation also supports the potential therapeutic strategy of inducing replication stress to selectively target ecDNA-bearing tumour cells [14, 20, 26].

While our study sheds light on ecDNA replication dynamics, it leaves open questions about the molecular mechanisms governing the initiation and regulation of replication on ecDNA. Whether ecDNA origin licensing follows precisely that of chromosomal DNA to fully avoid reduplication requires further investigation. If, consistent with early reports [24], ecDNA is replicated only once per cell cycle, its copy number can increase only through random segregation and subsequent selection, rather than through multiple rounds of replication in a single cell cycle [61]. Further, the roles of specific proteins or epigenetic marks in the altered replication timing and origin activation patterns remain to be elucidated.

Addressing these questions may be facilitated by our development of a new method for ecDNA isolation, FINE, which minimizes ecDNA processing and offers high specificity, potentially enabling the study of ecDNA properties such as chromatin composition and three-dimensional structure through proteomics and imaging. While FINE is limited by relatively low yields of ecDNA in large volumes and incompatibility with some replication assays like Repli-seq, it provides a potentially valuable tool for future studies.

Finally, while this study presents a detailed analysis of replication dynamics in two variants of a colorectal cancer cell line, ecDNA exhibits radical heterogeneity between cell lines and even among individual ecDNA molecules within a line. Therefore, future studies will need to examine the broader question of ecDNA replication patterns across primary tumour lines from a range of cancer types and ecDNA compositions. The application of advanced replication analysis techniques, such as DNAscent, and the improved ecDNA isolation methods we report here will allow a deeper understanding of the vulnerabilities in ecDNA replication, potentially allowing the developing targeted therapeutic interventions to control ecDNA copy number.

## Supplementary Material

gkaf711_Supplemental_Files

## Data Availability

Sequencing data can be accessed at the Gene Expression Omnibus archive with the accession number GSE186675. The code for the ini-seq 2 origin caller can be found at https://github.com/Sale-lab. The ONT sequencing data can be found on ENA under the accession number PRJEB83636. The code for the DNAscent-associated analyses is publicly available under https://github.com/Pfuderer/ecDNA_replication_dynamics and on Zenodo at https://doi.org/10.5281/zenodo.15792133.

## Notes

Present address: Oxford University Hospitals NHS Foundation Trust, John Radcliffe Hospital, Headley Way, Headington, Oxford OX3 9DU, United Kingdom

Present address: Fondazione Italiana Fegato, Area Science Park, Basovizza, Trieste 34149, Italy

Present address: Department of Medicine, Università degli Studi di Udine, Udine 33100, Italy
